# Correction: Strong inter-population cooperation leads to partner intermixing in microbial communities

**DOI:** 10.7554/eLife.02945

**Published:** 2014-04-16

**Authors:** Babak Momeni, Kristen A Brileya, Matthew W Fields, Wenying Shou

Momeni B, Brileya KA, Fields MW, Shou W. 2013. Strong inter-population cooperation leads to partner intermixing in microbial communities. *eLife* **2**:e00230. doi: http://dx.doi.org/10.7554/eLife.00230. Published 22 January 2013

In Equation 3, the cross product was an error introduced during typesetting and should be replaced by a dot product.

The article has been corrected accordingly.

Incorrect equation:

$$\frac{\partial S}{\partial t}=\nabla \times (D\nabla S)-U+Q$$

Correct equation:

$$\frac{\partial S}{\partial t}=\nabla \cdot (D\nabla S)-U+Q$$

